# Carcinoma

**Published:** 1882-09

**Authors:** L. B. Wells

**Affiliations:** Utica, New York; Bureau Clinical Medicine, I. H. A.


### CLINICAL BUREAU.

### CARCINOMA.

L. B. WELLS, M. D., UTICA, N. Y.

(*Bureau Clinical Medicine, I. H. A.*)

It is not the design of the writer in this paper to discuss the etiology and characteristics of the different forms of cancer, as developed in the various tissues of the body, but to impress upon the minds of his colleagues the propriety, in all cases, of ascertaining the exact simillimum, and prescribing with the expectation of at least relieving the patient's sufferings, even in its advanced stages, and in the earlier stages, of retarding its development, or arresting it entirely.

Too often have our friends turned off these forbidding cases to the surgeon or some specialist, under whose treatment with the knife or cautery a fatal result has been precipitated.

By a careful individualizing of each case, with the selection of the appropriate remedy administered at sufficient intervals, we may be assured that we are doing that which is for the best good of our patients.

An important question arises. Can scirrhus tumors be arrested in their growth, or entirely dispersed by the administration of the infinitesimal dose? It is time that skepticism on this matter should be silenced.

The physician should exercise a marked degree of patience and perseverance in all such cases, and not fail to impress the same upon his patient.

I will select from my case books a few cases.

Mrs. L. W. P., aged 50, consulted me for a tumor on the inner side of the left cheek, about the size of a dime, of a bluish color, causing an elevation on the outer side. The pains were stinging and burning.

A few months afterward another similar tumor was developed on the other cheek. In consultation afterwards with Dr. B. F. Joslin, Sr., it was decided that these were of a scirrhus nature.

At intervals of three or four weeks, in accordance with the symptoms, she had Sil.$^{200}$ and CM, Ars.$^{200}$ and Lyc.$^{200}$. After six years' persevering treatment, both the tumors with the concomitant symptoms entirely disappeared.

Although in other respects an invalid, she lived to the age of 81 years.

Mr. L. P., aged 72 (1872), had a hard, crusty tumor on the neck near the ear. Pains burning and twinging. He had at intervals Apis$^{30}$ and Lapis$^{30}$, but no improvement resulted. Ars.$^{200}$ at long intervals, with an occasional dose of Sil.$^{200}$, resulted in the removal of the tumor in two years. Two other similar developments on the face have also disappeared under the use of the same remedies. He is now 82 years of age and in good health. This is a case of hereditary diathesis, for two of his brothers died of cancer of the face.

B. F. J., aged 69 years. In June, 1869, had a crusty tumor on the lower lip, attended with burning and stinging pains. When the pains were burning and stinging, Ars.$^{200}$ gave relief. This was repeated over in three or four weeks, and with an occasional dose of Sil.$^{200}$. In one year the whole disappeared, and he is now living and has had no return of the trouble. A surgeon had informed him that nothing but the knife would relieve him.

J. B., aged 65 (1873), had a hard, uneven tumor on the inside of lower lip of the size of a three-cent nickel coin. Five years before had a similar one same location, apparently removed by caustics. The pains were stinging and the tumor had gradually increased during the past year. Sil.$^{200}$ once in two or three weeks resulted in its complete removal in one year and no return at this time.

MAMMARY TUMORS.—Mrs. A. S. R., aged 42. Sept. 20th, 1877. Five months since discovered a tumor on her breast about the size of an ordinary peach, somewhat irregular in shape and hard, attended with burning and stinging pains. Carb. an.$^{30}$, and Apis$^{30}$, were given without any perceptible effect, except to relieve the pains, but increasing continually in size. She then had Ars. iod.$^{30}$, three or four powders, and S. L. one week, with directions to repeat the Ars. iod. when there was a return of the pains. In two years the tumor diminished to about one-third of its size. If from any cause there is any pain or uneasiness in the breast, a few doses of the remedy will relieve. Was informed a few days since that the lady is now in good health, and she thinks that all trouble in the breast is removed.

Mrs. W. G. C., aged 61. Oct. 6th, 1879. For some three months has had frequent sharp, stinging and burning pains in her left breast, and on examination she discovered an oblong hard tumor of the size of a butternut. She had Ars. iod.$^{30}$, repeating occasionally, until the pain ceased. On return of the pain, the remedy was again taken as before with complete relief. April 10th, 1882, examined the tumor, which was about the size of a chestnut, soft and free from pain.

If space would warrant, I could give many others with similar results. I would earnestly persuade my colleagues to treat these cases with the same confidence they do other diseases, and not turn them over to a surgeon who would resort to the knife or cautery.

